# Improvement in Psychodynamic Psychotherapy for Depression: A Qualitative Study of the Patients’ Perspective

**DOI:** 10.3390/ijerph17186843

**Published:** 2020-09-19

**Authors:** André Løvgren, Jan Ivar Røssberg, Eivind Engebretsen, Randi Ulberg

**Affiliations:** 1 Division of Mental Health and Addiction, Oslo university hospital, P.O. Box 4959 Nydalen, N-0424 Oslo, Norway; j.i.rossberg@medisin.uio.no; 2 Institute of Clinical Medicine, University of Oslo, P.O. Box 1171 Blindern, 0318 Oslo, Norway; randi.ulberg@medisin.uio.no; 3 Institute of Health and Society, University of Oslo, P.O. Box 1130 Blindern, 0318 Oslo, Norway; eivind.engebretsen@medisin.uio.no; 4 Department of Psychiatry, Diakonhjemmet Hospital, Diakonveien 12, 0370 Oslo, Norway

**Keywords:** improvement, depression, psychodynamic psychotherapy, patients’ perspective, qualitative study

## Abstract

The patient’s perspective on improvement in psychotherapy is crucial for tailoring the therapy he or she is receiving. The present study aimed at exploring the factors aiding and the patients’ experiences of improvement in time-limited psychodynamic psychotherapy for depression. Semi-structured, in-depth interviews were conducted with ten adult patients who received up to 28 sessions of manualized psychodynamic psychotherapy in the Norwegian study “Mechanisms of change in psychotherapy” (the MOP study). The post-therapy interviews addressed the participants’ experiences from therapy. The data were analyzed with thematic content analysis and hermeneutic interpretation. The analysis identified four helpful dimensions: “Therapist activities” comprised supporting and acknowledging, advising and offering tips for everyday life, questioning and pressuring. “Patient activities” included opening up, caring for oneself and showing agency. “Facilitators” for improvement were learning from therapy, learning to receive therapy and agreed goals. “Achievements” comprised new perspectives and understandings, increased self-awareness and mastery and changed thinking and feeling. Improvements from psychodynamic therapy seemed reliant on the degree to which the therapy could activate and be relevant to the patients’ everyday life. Tailoring therapy for patients with depression should link the focus on symptoms and ways of thinking and feeling with their life circumstances.

## 1. Introduction

Depressive disorders are the third leading cause of disability worldwide [1]. In spite of the increased use of antidepressant medications and easier access to modern psychotherapy, major depressive disorder (MDD) is expected to rank first in terms of Disability Adjusted Life Years (DALYs) in high-income countries by 2030 [2,3]. MDD is associated with significant mortality, economic burden, reduced quality of life (e.g., impeded relations in family or social life) and diminished capability to work or study [4].

Although medication and psychotherapy are helpful for many patients, they do not help everyone, and many patients relapse and suffer from repeated depressive episodes [5]. Some patients even experience deterioration or worsening in therapy [6,7]. Randomized controlled trial studies (RCT) often overlook patients’ subjective experiences, and there is a need to improve psychotherapy to better help depressed patients. To achieve this, it is crucial to understand how patients experience therapy and what they identify as helpful.

The body of qualitative research addressing these questions has grown in recent decades [8,9,10]. This has led to several meta-analyses across psychotherapeutic orientations, influenced by the works of Ladislav Timulak [11], who reviewed seven studies and identified several helpful features across therapies, including new insights, emotional experiences and behavioral strategies; the acceptance and understanding of the therapist; involvement in treatment; and a human connection to the therapist as part of a supportive, safe relationship. Another meta-analysis included 41 studies that focused on patient-identified significant moments in psychotherapy. The findings were highly similar to those of the first meta-analyses, and the main findings were new insights, awareness and problem resolution along with feeling understood and reassured [12]. Insight was central in both of these meta-analysis. Timulak and McElvaney [13] conducted a meta-analysis of seven studies on significant events leading to insight. Their analysis revealed two types of insight: 1) Painful/Poignant insight, where clients realized something that was painful, including feelings of sadness, upset and hurt. The therapists’ empathic reflections or collaborative interpretations facilitated these events. 2) Self-Asserting/Empowering insight, where the therapists’ supportive and validating reframing promoted positive experiences and facilitated these events.

These meta-analyses foregrounded a comprehensive qualitative meta-analysis on clients’ experiences of individual psychotherapy. Levitt et al. [14] built on the works of Timulak and colleagues and reviewed 109 studies, grouping their findings into five clusters with numerous subcategories: (1) Therapy is a process of change that involves structuring curiosity and deep engagement in pattern identification and narrative reconstruction. (2) Caring, understanding and accepting therapists allow clients to internalize positive messages and enter the change process of developing self-awareness. (3) A professional structure creates credibility and clarity but casts suspicion on care in the therapeutic relationship. (4) Therapy progresses as a collaborative effort with a discussion of differences. (5) Recognition of the client’s agency allows for responsive interventions that fit the client’s needs [14].

However, comparing qualitative findings from different studies from a variety of contexts could weaken the significance and uniqueness of each study when abstracted, summed up or in other ways made manageable in ways more associated with a quantitative research paradigm [15]. This is addressed by Levitt et al. [16] for the case of qualitative research on client experiences of psychotherapy. The agendas of the researchers, the context and circumstances under which the qualitative studies took place, the identities of the participants and what questions they were asked are of great importance when interpreting, learning from or comparing the findings [17]. As the same helpful factors may mean different things in different patients’ lives, there is a need for studies focusing on the meaning attributed to the experiences and how the patients contribute to the therapy process [18]. The present study contributes to this by exploring how ten adult patients suffering from depression experienced, and attributed meaning to, improvement in manualized time-limited psychodynamic psychotherapy (PDT) with sampling control, a check of treatment fidelity and therapist supervision.

Qualitative studies restricted to psychodynamic/psychoanalytic therapies have explored different aspects of helpful in-session factors. One of these studies, included in the abovementioned meta-analysis of Levitt et al. [14], found that for young adults the therapeutic relationship and the therapist’s way of working, continuity and overcoming obstacles were helpful [19]. One study not included in the meta-analysis found that patients felt it was helpful to work through obstacles, such as a strained relationship with the therapist [20]. Others have found a secure attachment to the therapist, understanding of interpersonal and emotional patterns (insight) and restoration of a coherent life story to be helpful factors [21]. Werbart and Levander [22] reported the importance of working through the patients’ unrealistic wishes for what to achieve through therapy. Factors such as sharing what is inside of oneself, gaining new perspectives and understandings and therapy as a place and time for oneself have also been found to be helpful [23]. These studies also seems to fit with the clusters identified in Levitt et al. [14], although the study designs and selection of the participants varied. None of the studies examined time-limited PDT, and this well-established and widely used routine therapeutic intervention has been the subject of little qualitative research.

To the best of our knowledge, only one previous qualitative study [24] has examined adult patients’ experiences of time-limited PDT for depression with a study design comparable to that in the present study: sampling control, manualized treatment, therapist supervision and treatment fidelity control according to the theoretical underpinnings of the therapy. De Smet et al. [24] interviewed improved and recovered patients in an RCT study on cognitive behavioral therapy (CBT) and time-limited PDT for depression. The study also found that the patients improved and recovered when they cared for themselves, gained increased self-understanding and learned new coping skills. The study included improved patients from both the CBT and the PDT groups according to self-reported depression symptoms.

Rigorous study designs, such as in the present study and in the study of De Smet et al. [24], are important because they strengthen the probability that the therapists actually delivered the intended therapeutic intervention. Understanding the experiences of even a few patients, as in the present study, has the potential to advance the theoretical understanding of the change processes and increase the effectiveness of psychotherapy [25]. Insight into the patients’ experiences of psychotherapy might inspire and contribute to therapeutic work with other patients—the main aim of the present study. Accordingly, the present study explored how patients participating in a large RCT study on depression experienced improvement in time-limited PDT and the significance of their experiences.

## 2. Materials and Methods

The study took place within an ongoing RCT on mechanisms of change in psychotherapy in Norway called the MOP study (Clinical trial registration: Clinical trial gov. identifier: NCT03022071). By experience, we refer to the thoughts and feelings the patients communicated about their therapy in the interview situation. Hence, we limit experience to the patients’ retrospectively verbalized feelings and thoughts about their therapy, conveyed during a research interview under particular circumstances. However, non-verbalized or pre-reflexive experiences from therapy may be just as real and influential to the patients, even though they did not verbalize them in the present study.

### 2.1. Design, Ethics and Data Collection

The Central Norway Regional Ethics Health Committee (REC South East 2016/340) approved the study. Oral and written informed consent was obtained from all participants, randomized to 16 sessions and three monthly booster sessions of CBT or to 28 sessions of PDT. Inclusion and treatment in the MOP study is still ongoing. Hence, outcome data for the participants are not available. When the patients finished the treatment, they took part in a follow-up evaluation. The evaluators invited all patients who met for this evaluation in a given time period (*n* = 12) to participate in an explorative, qualitative in-depth interview. The open, though semi structured, interviews had a duration of 45 to 60 min and took place at the outdoor clinic where the patients had received therapy. We aimed for an informal and supportive tone in the interviews, encouraging the participants to elaborate on themes of relevance. The questions covered the patients’ experiences from therapy in general, what had been helpful or not so helpful, how therapy had affected their relations in life outside therapy and the extent to which the patients could use anything from therapy in life outside of therapy. The second author, who was also the leader of the MOP study, conducted the interviews. A research assistant transcribed the interviews verbatim and anonymized all the transcriptions.

### 2.2. Participants

Of the 12 interviewed participants, two had dropped out of therapy due to dissatisfaction. They reported no experience of improvement and were not included in the present study. Of the remaining ten, one completed 18 sessions, while the others completed all 28 sessions. The mean age at inclusion of the interviewed persons, seven females and three males, was 32 years (range 20–45). All met the criteria for MDD according to the Mini International Neuropsychiatric Interview [26]. The mean baseline level of depression was 17 (range 11–26), as measured by the Hamilton depression rating scale [27]. Four patients were diagnosed with personality disorder according to the Structured Clinical Interview for DSM-IV Axis II [28]. One was unemployed; the rest studied or worked, but sick leave did occur. Five lived with a partner, while the others lived with parents, alone or in other ways. Several had children.

### 2.3. Therapists and Treatment

All therapists had a minimum of two years training in PDT. In addition, they received one year of training on the principles of time-limited PDT before receiving study patients for therapy. Experienced senior PDT researchers/clinicians monitored adherence to the treatment principles through weekly group supervision of the therapists, including discussion of all the therapies, throughout the therapy period. The supervision was based on video recordings from the therapy sessions and focused on the initial phase of treatment, establishment of a case formulation, how to conduct treatment for the individual patient and termination of the time-limited treatment. The dynamic therapy was based on the general psychodynamic principles described in Gabbard [29]. Short-term psychodynamic psychotherapy (STPP) is a well-known treatment mode based on general psychodynamic principles, including interpretation of unconscious conflicts, insight and the concepts of internal working models. The STPP manual used in the “First Experimental Study of Transference-Interpretation” [30], describing specific features of time-limited therapy, was used as the guideline for treatment. Further, the treatment encouraged the patients to explore sensitive topics, including an exploration of the patient–therapist relationship [31]. The therapists also focused on interpersonal relationships outside of therapy as a basis for their interventions.

### 2.4. Analysis

The analysis process and the interviews proceeded simultaneously, and the interviewer gained knowledge and experience as more interviews were conducted. This affected the interviewers’ reflections on and awareness of the subjects and topics brought up by the participants and informed the final analysis [32,33,34]. The first author read the transcribed interviews guided by the following question: “What do the participants say about improvement?” This allowed familiarization with the data, provided a first impression of the themes and subthemes and reduced the totality of the transcribed material for further analysis. The first author then read and re-read the texts several times and identified themes and subthemes that best represented the stated meanings, viewed in relation to the text as a whole. The second author, who conducted the interviews, gave feedback and commented on the coding into themes and subthemes. This inductive thematic content analysis did not attempt to fit the data to pre-existing concepts or theoretical ideas [35]. All authors read, commented on and contributed to the analysis as well as to the manuscript as a whole. This approach strengthened the internal validity of the findings and made the interpretations less dependent on individual preferences.

Our analysis used a hermeneutic approach [36], according to which understanding is a continuously interpretative process where the interests and pre-understandings of the interpreter—the horizon—is tested against the text. The horizon is “(…) an opinion and a possibility that one brings into play and puts at risk, and that helps one truly to make one’s own what the text says” [36] (p. 406). Like the interviewer is not on the outside of the interview, the interpreter is not on the outside of the text but is rather a part of it and becomes engaged with it through conscious and unconscious preconceptions. For example, the first author’s preconception was influenced by not being a psychotherapist but an experienced nurse from the field of forensic psychiatry. The co-authors were two psychiatrists trained in CBT or PDT and one historian of ideas.

To understand something, then, means to activate the preconceptions, not to ignore them, as it is not possible to get rid of them and still achieve a clear understanding of something in the world “out there” [37]. Often referred to as the hermeneutic circle, our interpretations of a part of the text influenced the interpretations of the larger part of the same text. This, in turn, affected and sometimes changed the interpretation of this part in an ongoing circularity of part–whole interpretations [38]. This process also included text fragments of what the interviewer said during the interviews. The interviewers’ questions and responses also affected the best way to interpret and understand the patients’ utterances.

## 3. Results

The analysis identified many actions and processes within the therapy sessions that led to improvement. These were categorized into four dimensions with several subthemes (see Table 1). They were not clear-cut, and the same text fragments could illustrate more than one theme or dimension. The first dimension refers to the therapists’ activities and comprised supporting and acknowledging, offering advice and tips for everyday life and questioning and pressuring. The second dimension describes the patients’ activities and included opening up, caring for oneself and showing agency. The third dimension refers to the facilitating factors in therapy and comprised learnings from therapy, learning to receive therapy and having agreed goals. The fourth dimension—achievements in therapy—included new perspectives and understandings, increased self-awareness and mastery and changed thinking and feeling. The patients experienced several of the same factors as helpful. However, there could be large variations among the patients in terms of how they emphasized these factors and the meanings they attributed to them. The results were ordered by what the participants regarded as most helpful, what the authors saw as most striking in each patient’s story of improvement and by the meanings different participants attributed to their experiences. The quotes that are given below illustrate the themes presented, but they do not necessarily reflect the actual patient’s most distinguished experience from therapy.

### 3.1. Dimension One: Therapist Activities

#### 3.1.1. Supporting and Acknowledging

The patients could feel supported and acknowledged in two ways. One occurred when the therapist gave direct praise or used non-critical words to describe one patient’s suffering. This was done as an alternative to the patient’s own harsh words: “*That someone else know and listen to how I am, without being as critical as me, one who sees that I’m suffering but puts other words to it than I would have done*”. This was helpful to the patient and made her feel that the therapist really saw her. The therapist of another patient said that things would be all right. Due to the patient’s varying receptivity, the therapist repeated encouraging platitudes and sayings. This helped the patient to gradually accept herself and integrate the hope she felt the therapist gave her: “*She said she cared, and I got the feeling that she saw the whole of me and the sides I didn’t like (…) She held on to that it was possible to fix me, that something within all the chaos was of value*”. The patients could also experience support and acknowledgement when the therapists normalized the patients’ situation, thoughts or feelings. In addition, they could experience support and acknowledgement simply when the therapists listened to them. For one patient, being listened to caused her to feel that she was being taken seriously by the therapist and that her problems, especially those from her childhood, were not because of her: “*She listened and like, in a way, confirmed that it shouldn’t be like that. I think she just made me talk and talk until I realized it wasn’t my fault*”.

#### 3.1.2. Offering Advice and Tips for Everyday Life

The patients valued receiving tips or suggestions about what to say or do to master everyday challenges. The importance of talking about such challenges and difficulties was a central experience. One patient illustrated this by what she actually chose to talk about in therapy when she was given the choice: “*You in a way could talk about anything. And I talked about a dinner party where I didn’t manage to talk because I was so afraid of saying something stupid or something like that*”. Another patient experienced the concrete and everyday orientation in this way: “*I went through the week and then I could stop at things I’d like to talk about*”. Some of the everyday problems appeared in close relationships, while others were due to strained thoughts and feelings, and therapy offered a place to practice difficult tasks: “*Totally, what have been the most important to me is that I’m actually talking with my wife about how I feel. I now have some practicing in talking about difficult things and feelings*”. Another put it this way: “*What my therapist made me do was to put myself into focus in an everyday in which I felt I couldn’t put myself into focuses*”. For another patient, the focus on the concrete was what she needed to improve: “*Sleep, and go out and do things. Because I almost just sat inside watching TV-series all day*”. Another patient improved by working on her relational challenges from life outside of therapy within the therapy sessions. She believed the therapist recreated and challenged these relationships and, in one moment, she even wondered whether or not the therapist liked her. This, however, made her more conscious of herself at a deeper level, which led to a personal revelation: “*I just have to trust myself. I can’t be liked by everyone*”. She contrasted this experience with her expectations for therapy, thinking her problems were due to her negative thoughts about not being good enough.

#### 3.1.3. Questioning and Pressuring

The therapists asked different types of helpful questions that served several purposes. A central question was the “why” question. This challenged and helped the patients to really think through and explain their opinions about themselves, other people or a situation. Often, they realized that these beliefs and judgements were wrong or that their ways of thinking did not make sense or were unreasonable. One patient expressed how the therapist’s “why” question had helped her: “*’Why’. She* [the therapist] *was rather stubborn. She kept pressing me to do things, or questioned things I not at all felt any need to questioning; ways of thinking or behaving, just everything. So ’Why’*”. Helpful questions could be confrontational or they could help to analyze a demanding situation. They could help patients to not remain stuck in depressive feelings: “*Instead of thinking that this is shit, so just: ‘why do I feel like this? How did it become like this? What can I do with it?’*” Some questions, which had a more existential character with a focus on what the patients really wanted to do in life, helped them to make other choices and gain new experiences in their everyday lives. An important aspect of questioning was the way in which the therapists asked their questions: “*It’s the way she responds, the way in which she asks or continue the conversation that creates trust*”. The therapist’s nonverbal communication style and the emotions the therapist signaled when asking the questions were also central: “*Then she, some laid back, said: ’Does it matter, anyway?’ And I thought: ’Well maybe it doesn’t’*”. One patient improved and gained more self-confidence through better contact with her feelings, achieved by exploring questions: “*It was her questioning. It could be ’What do you feel now?’ A question simple as that*”. Another patient improved by exploring her worries and the negative fantasies she had about the way others would view her: “*She asked questions like ’Yes, but what would had happened if you had said something? Would people (…) what (…)?’ She almost repeated it all the time: ’What had happened?’ only to illustrate that it isn’t so much that might happen, it isn’t that much*”. This helped her to free herself from her worries and to focus more on the person she was: “*It was something with letting others’ thinking go, others’ thoughts about me go. And rather focusing on the core of myself*”.

### 3.2. Dimension Two: Patient Activities

#### 3.2.1. Opening Up

For some patients, a basic feature of opening up was actively choosing to be open with their therapist and with themselves. This came more naturally to some than to others. The patients experienced strain or made great efforts related to openness, but they improved when they managed to open up about their feelings and the situations they had dealt with. One patient emphasized that choosing to be open was essential for her improvement: “*I think it was a combination of some trust and that I kind of decided for myself that I wanted to become better. And I believe the only way to be better is to open up oneself*”. Another patient, who found it difficult to be open to others, summed up what she felt had been helpful in her therapy: “*It just helped that I could open up myself a little. Be honest and just say things as they are and then, in a way, being heard. I felt I could be open without being deemed*”. As she became more open in therapy, she realized that she was usually not very open with others. Another patient put it this way: “*It’s kind of a relief just putting words on it. Then it’s not something just on the inside of you*”. One patient was not used to being open about her feelings, and she did not see the benefits of being open with the therapist either. However, in retrospect, she realized it after the therapy had terminated: “*It was that we talked much about the same things. But, as I somehow feel, it was a reason for it*”. A male patient reported that his openness in therapy was a way to practice talking about difficult themes. This helped him to be more open with his partner. He improved through and appreciated the trust he felt in his therapist due to the way the therapist responded to his openness: “*She was very good at telling me how she understood me, if she got it right. That was very good and surely increased the trust. All the time I knew she got it right, and didn’t misunderstand or misinterpreted anything*”. Another male patient improved by opening up about what he really felt and not just talking about what he felt superficially, as he used to do: “*I managed to open up. I’ve always been thinking I’ve been very open about my feelings because I could talk about them. But I don’t think I was*”.

#### 3.2.2. Caring for Oneself

The patients improved in two ways when they cared for themselves: outside of therapy and within the therapy sessions. Outside of therapy, the patients improved when they took better care of themselves instead of accepting what was happening to them. These changes occurred when the therapists helped the patients to see that challenging situations or relations could be changed. With the therapists’ encouragement and support, the patients discovered that they themselves could have an impact on their circumstances: “*We talked a lot about this. It strengthen me and I feel today that I in some way see the value of it. Now I take time for myself in which I shall love myself and take care of myself*”. The patient even saw this self-care as her way to avoid relapses: “*I think I will use this for the rest of my life to prevent depression*”. Another patient felt she did not gain very much in therapy and completed 18 sessions. Nevertheless, she appreciated the focus on taking care of herself by saying no to things, being aware of her wishes and needs and communicating them to others: “*Now I’ve tested it* [to speak up]*. It’s not the end of the world, one does not necessarily get rejected, which is positive. So it’s just to keep going and practicing it*”. Another self-care experience was to stop thinking negatively about oneself. One patient complained about his partner, but the therapist made him realize that he could affect the relationship: “[the therapist] *’Yes, she sets the agenda, but it’s also up to you setting the agenda’. And I, like, ’okay, that’s true’. A person might set many premises but you yourself also have to set the agenda as the relationship is to be run by both. This became a little revelation*”. Another patient was relieved to discover that he could view himself as an actor with agency and act more clearly towards his partner: “*It was something with that power and impact, and not having to feel so disheartened and alienated*”.

#### 3.2.3. Showing Agency

Within the therapy sessions, the patients improved when they showed agency toward the therapist. One patient felt that therapy did not help her, and on two occasions she explained this to the therapist. The first time was due to having the wrong focus: “*Just saying* [to the therapist] *’Do you know what, this is not going forward’ helped me. I felt we were working with some inner self and I was whatsoever not there at all. I hardly could get up in the morning*”. The second time she felt that she had a long way to go and needed the therapist to not give up on her: “*When I got the response the first time and things actually got better I felt like ’It’s possible to speak up’, and that gave some mastery. When you’re down and depressed you don’t feel any mastery*”. By confronting the therapist, she changed the direction of the therapy, experienced mastery and improved. One patient did not know whether the therapist liked her (a question from a questionnaire she completed during the research project). She addressed this with her therapist, and they talked about it for one session. Consequently, she felt that she reached a turning point in her relationship with the therapist: “*She* (the therapist), *like ’Do you think I like you, or do you think I don’t?’ It became rather confronting but I felt it somehow got better, then, when I, like, addressed this and we focused on it*”. Another patient erected boundaries and avoided asking the therapist about personal issues. This was something she initially thought might help her to build a relationship with the therapist: “*It somehow was okay just talking about myself. I feel that if I had another relation to her it somehow would have been more difficult talking to her*”. She prioritized her own needs by not giving in to her impulse to focus on the therapist.

### 3.3. Dimension Three: Facilitators

#### 3.3.1. Learnings from Therapy

The patients tried to remember things the therapist had said or done and apply them to their everyday lives. In particular, the “why” questions were helpful, and the patients improved by transmitting these questions to life outside of therapy: “*I use it very often when I’m stressed, or worried, like ’What might happen, and why is it so bad?’*” Others used concepts like “strategies” or “tools” to explain how they used learnings from therapy as a way to manage symptoms or difficulties outside of therapy. Another learning was to give oneself rewards when doing something good: “*I thought the only way to learn something was to punish myself. I always punished myself, but that we turned around*”.

#### 3.3.2. Learning to Receive Therapy

The patients underscored the importance of being familiar with how therapy works and how the therapist would conduct the therapy. One patient improved and reached a turning point in therapy when, after approximately ten sessions, the therapist made it clear that she would gain more from therapy if she also gave more of herself: “*That was a turning point. I was open for therapy but not open for contributing. I was very open for that therapy should make me better and such things but I wouldn’t participate in it, if you see, because I thought it was so difficult*”. Another patient gradually developed a better relationship with her therapist. She improved when she changed her incorrect expectations of therapy as a place to talk about everything from childhood and began to understand how she and the therapist were supposed to work together. Initially she experienced the silence in therapy as the therapist’s lack of interest in her. However, she learned that the silence had a purpose: “*I think it was, like, to be familiar with the therapy and that she just sat there and listened to what I said (...) After a while it also became comforting; that it’s possible to be silent and thinking through what has been said*”. One patient had to learn what getting familiar with the therapist meant. She asked the therapist personal questions, felt dumb and became uncertain. Nevertheless, she experienced some advantages from the therapy work when she did not know much about her therapist she dealt with: “*It became more neutral, and was easier to go into those challenging relations*”. Some patients indicated that the therapist explained what methods they would use, and that silence was part of these methods. This made therapy more predictable. Once the significance of silence was understood, it was experienced as effective and helpful, as indicated by this patient: “[the therapist] *like forced me to take control, to take initiative and to talk about things.* [The silence] *just forced out some fighting spirit. A bit silly but it forced a lot out of me*”. Another experienced the silence in this way: “*It triggered me to think*”. Yet another patient recognized the value of the silence in retrospect: “*Sometimes it was frustrating, other times it was good. In retrospect, it surely was good because I myself had to think and find an answer*”.

#### 3.3.3. Agreed Goals

The patients attributed different levels of significance to having goals in therapy. One patient improved gradually through therapy, became less angry and explained the connection between goals, feelings and mastery: “*I felt a lot of anger, and I noticed that this pressure was reduced. I believe this came about as we established some new goals. Constantly looking at the goals and reevaluate them, like* ‘*You know what, we’re far away from that’ or ‘Wow, this we’ve managed, we can move on’. Because when you are down you don’t feel any mastering. Then it’s important to have goals that you master, things you manage to do*”. Another patient reported that beginning to work again had much greater significance to her depression then she initially was aware of, adding: “*We also sat three goals, which were to free me from acknowledgment from my parents, become more self-secure and be more creative*”. Setting goals was also significant for another patient because she was dreading the sessions, feared she would not be taken seriously and wondered whether she really had good reasons for being in therapy: “*My therapist took things very seriously, and we defined a kind of main goal in the therapy. At once it became, like, ’okay, there is actually a reason for being here’. I became convinced*”. The patient experienced that she was met in a serious way through agreed goals, which also served as a validation of her suffering.

### 3.4. Dimension Four: Achievements

#### 3.4.1. New Perspectives and Understandings

The patients gradually achieved new or more nuanced opinions about themselves when they realized the limitations of their original opinions: “*I do think a lot about myself. But what I think isn’t necessary what other thinks*”. For one patient, receiving other opinions was related to hope and motivation to make changes: “*Once a week going to a person and emptying the garbage. At least it gave me another view and more hope. More guts, like ’Okay, I’ll fix this!’*” New perspectives could also come about through the therapists’ open questions: “*He just said, like, ’Why haven’t you looked at it from another side? There are more sides’. Then it became, like, oh my God, so ridiculous. Everything has more than one side. Always*”. Other patients obtained new perspectives through the therapists’ interpretation or feedback: “*She had some comments or did some observations from what I said, and made me see things in a different way. She was good at, like ’So, what you mean are (…)’ or ‘what you say are (…).’*” New perspectives could also arise simply through the therapists’ affirming and supportive way of being, which for one patient contrasted previous negative experiences of close relationships: “*In a way she was an opposite of all those patterns I’ve build, an opposite of bad experiences. She became an opposite of how I believe other relates to me*”. The patients underlined how important trust and confidence in the therapists were for integrating new perspectives. One put it this way: “*He just couldn’t had done it, say, after two weeks and, like, ’Yes, but look here (…)’. Then I wouldn’t had believed it*”.

The patients achieved new understandings during therapy. This could mean understanding something they had not been aware of before or a new perspective on something they had already known. One patient gradually came to understand the circumstances in her childhood that had made her too independent as a child and too analytical and practical as an adult: “*I began to see the connection a lot more, see my story a lot more; how I became the one I am: not good at being vulnerable*”. A new understanding of a troubled family background led another patient to more nuanced opinions about himself and his depression: “*It created some kind of understanding; that things have had their reasons (…) and realizing for myself that this is a disorder, it’s not my fault*”.

#### 3.4.2. Increased Self-Awareness and Mastery

For some patients, increased self-awareness related to the existential dimensions of life. One patient had a troublesome relationship with her partner and entered therapy thinking she should fix her depression and thereby her relationship. That did not happen: “*In the process of going to therapy, you became more aware of who you are. What your needs are, what you can live with, and what you can live without*”. She had to break up with her partner, which she explained as follows: “*When being more aware of myself and coming out of the depression, you see that you’re more worth than you think*”. For another patient, increased self-awareness related to the existence of life itself—and what to do with it: “*Theoretically I just may take my own life without anyone to stop me. Not that I think of that now but I think much of my troubles was a fear of, like... ’I can do whatever I want. Where shall I begin with all this?’ (…) There were so many questions I didn’t manage to bear. What was important to me?*” Working through these existential questions helped her to find her own way. One patient described what had been helpful in therapy for her: “*I had a room to open up and room to see myself*”. This achievement occurred due to the therapists’ feedback and descriptions of her: “*She could see things from the outside and offer quite good descriptions of me, which I hadn’t thought of before. I kind of became more aware of myself*”. An opposite way to achieve self-awareness was demonstrated when the therapist of another patient drew some connections to the patient’s past. The patient became more aware of himself through contrasting his difficulties in life today with an earlier and challenging period of his life, specifically when his parents divorced: “*Those things were in a way waked up again; that of restraining my problems and not telling about them. Such parallels that (…) It kind of raised awareness and made me see myself at that time and that I don’t want to go in that (…)*” For him, the focus on the past and his increased awareness of who he was today was a turning point in therapy.

#### 3.4.3. Changed Thinking and Feeling

Changed thinking was a salient feature of improvement, sometimes closely connected to silence: “*In the silent periods you kind of could reflect a little more*”. Changed thinking could relate to accepting ones’ situation: “*In one way I still think in the same way. But it’s like I’m aware of other ways of thinking; that everything isn’t dark all the time. Like, it’s okay to be where you are in life even if you don’t want it, it’s just how things are now. Just accept it*”. Changed thinking also related to reduced rumination: “*To let things go. I didn’t have to ruminate that much: Why I’m so stupid, why I’m so quiet, why I don’t get along with people, why (…) It circulated (…) Why am I like this?*” Rumination about personal matters was one of this patient’s most extensive symptoms, and changing these thoughts led to the most improvement in therapy. Another, male patient was able to reduce his rumination by changing his focus from situations to emotions: “*It’s about those ruminations. That you rather should go into the feeling and not the situation. If I feel anxiety or tension, then rather stay in it and feel it. Instead of trying to find a solution to the situation*”.

Changed feelings occurred in several ways, and the patients could feel too much or too little, have incorrect feelings or not know their feelings well. A focus on feelings was a central theme in the patients’ improvement: “*I practice on being angry. I went from being only indifferent or sad to a wider emotional range including some positive feelings. I think both positive and negative feelings are difficult*”. One patient did not achieve a change in her feelings per se; rather, she *began* to feel: “*That I got some ease of my feelings as I could open up to her. I got a room to cry and to feel that thing hurts. When I suppresses my feelings and the pain (…) it anyway will be there. I suppose I brought it up the light*”. Another described it in this way: “*If I should mention a real trigger point then it would be to listen to my feelings and not just be in my head. This created the greatest change*”. One patient needed the therapist to express her own feelings: “*She* [the therapist] *expressed herself in a way so I could recognize my own feelings and better understand them*”. For one male patient, the change in how he related to his feelings was the most helpful achievement in therapy: “*What I think I gained most from in therapy was that I learned to open up and talk about feelings*”.

## 4. Discussion

The present study explored how adult patients experienced improvement in time-limited PDT. The analysis identified four main dimensions, categorized as therapist activities, patient activities, facilitators and achievements. Different studies conceptualize and group their findings in different ways, depending on, for example, the investigators’ preunderstandings. As a result, the concepts and categories developed in the present study are not necessarily specific for time-limited PDT. They may also be found to align with the results of studies of other psychotherapeutic interventions. Nevertheless, there seem to be several overlapping conceptualizations in the framework of the present study and the framework developed in the meta-analysis of Levitt et al. [14]. One important aspect, however, is the need to conceptualize and categorize individual patients’ experiences. Another is the meaning and significance different patients attribute to their experiences. The present study focused on the significance of the patients’ experiences with therapy as seen in retrospect, not on how therapy might have caused these experiences.

Different findings from different qualitative studies may reflect differences in the content of the therapies under study. Obviously, it may be difficult to experience something as helpful if it were not part of the therapy. Further, due to the researchers’ backgrounds and preunderstandings, differences in the reported experiences may also reflect variances in the conduct of the qualitative interviews and in the research designs. The present study, for example, was on supervised and manualized time-limited PDT for depression, making it challenging to compare it to studies of other therapies with different study designs.

### 4.1. Therapist Activities

In contrast to the psychodynamic/psychoanalytic studies of Leonidaki et al. [21], Palmstierna and Werbart [19] and von Below et al. [23], which mainly reported relational and intrapersonal factors as helpful, the present psychodynamic study found it was helpful for therapists to also offer advice or tips for everyday life. Practical factors were also reported for other adults [24], elderly individuals [39] and adolescents [40]. In Dakin and Arean [39], the relevance of practical orientation may partly be explained by the fact that their patients also suffered from executive dysfunctions. Nevertheless, as Bohart and Tallman [18] observe, patients tend to take from therapy what they need to get better. Hence, similar findings may indicate that patients in different studies enter therapy with coinciding needs that they seek to satisfy. Those needs are not necessarily known to the therapists or to the researchers. In any event, we view practical-oriented interventions as important in psychodynamic therapy, despite the fact that such interventions are “…classically regarded as not truly psychoanalytic” and are, theoretically, described as “…secondary strategies” [29] (p. 115).

Another helpful therapist activity was questioning, either by asking exploratory questions or by doubting and carefully framing the patients’ opinions through, for example, the “why” question. The value of asking questions was also highlighted in a study on relationally oriented therapy [41]. Improvement through questioning is a rarely reported helpful experience in psychotherapy. Perhaps questions are regarded as self-evident and are taken for granted in psychotherapy. Nevertheless, patients seem to gain insights and develop alternative ways of problem-solving through questions, especially the “why” question. In CBT, for example, the use of Socratic questions holds a prominent place in the “guided discovery” intervention [42,43]. In the present study, however, the patients emphasized the emotional and relational qualities that the therapists transmitted along with the questions. The helpful questions did not stand alone; that is, they were not only useful for making discoveries about themselves. Rather, the questions were embedded in what the patients experienced as trust in or comfort with the therapists. Asking questions entailed more than what the questions literally meant, and the patients seemed to attribute personal qualities to the therapists based on the way in which they asked their questions. Questions asked in an adequate and satisfactory way could give patients a feeling of being understood and of having value, conveying genuine interest and contributing to a good relationship with the therapist. For the patients in the present study, it seemed important that the therapists were aware of this emotional dimension of asking questions.

As in the present study, Levitt and Piazza-Bonin [44] found that, with different types of therapies (not only PDT), feeling pressure from oneself and the therapist and receiving guidance and approval from the therapist were central themes for improvement. Such support from therapists and help with everyday issues like relationships seem to resonate with findings from studies across the human life span. For example, studies of problem solving therapy (PST) and supportive therapy (ST) in elderly individuals [39], psychodynamic therapies in adolescents [40] and adults in the present study have all reported several of the same helpful experiences among patients in three different phases of life, with great variance based on development, age and social roles. The patients in these three studies seemed to have all experienced improvement in psychotherapy to the extent that the therapy, directly or indirectly, related to and had some positive effects not only on symptoms or personal well-being but also on their relations and life outside of therapy. This suggests that patients, independent of age and other circumstances, share some common conditions regarding what to achieve through therapy, which should be kept in mind by therapists meeting patients of different ages.

Some of the valued therapist activities, such as support and help with everyday issues, may be regarded as non-specialized and mundane themes that are common to most people’s lives. However, others have reported similar results [45,46,47,48]. As mentioned above, patients generally seem to take from therapy what they need to get better [18]. Emphasizing discussions about mundane or non-specialized issues as a helpful strategy in therapy—and the importance of an active patient—does not mean that the findings of the present study diminish the contributions and value of therapists. It also does not mean that the work of the therapists in the present study was simple, non-specialized or solely talk, as the present study did not explore what the therapists actually said or did. Rather, as one implication of the present study, the findings indicate we should not overlook how important “simplistic” or common themes may be for patient improvement just because they seem too obvious. Others have made similar arguments, reporting that eye contact, warm and personalized greetings from therapists and paraphrasing are crucial for alliance in psychotherapy [49].

### 4.2. Patient Activities

As in the present study, De Smet et al. [24] found that patients improved and recovered from major depression when they actively cared for themselves, increased self-understanding and learned new coping skills. The findings illuminate that patients in therapy are active contributors. De Smet et al. [24] included improved and recovered patients from both CBT and PDT groups in their analysis, measured by statistical outcomes. These measurements, however, did not grasp the multidimensional nature of the outcomes in the way the patients experienced them. For statistical measurements to be clinical meaningful, they should be interpreted and contextualized within the patients’ personal narratives. Several other studies have also shown that improvement, even recovery, goes beyond symptom relief, which is what is typically measured in outcome research [50,51,52]. The present study reports solely on PDT patients where the outcome measures are unknown due to the ongoing recruitment of participants. This makes the selection of participants less comparable to that in De Smet et al. [24]. The present study reports on the patients’ opinions and understandings of their improvement. Nevertheless, we argue for the importance of combing measured outcomes with experienced outcomes when evaluating and developing psychotherapy [53].

### 4.3. Facilitators and Achievements

The patients achieved new perspectives and insights during therapy, similar to what was reported in von Below et al. [23]. Together with changed thinking or feeling, this helped them to manage better outside of therapy. Furthermore, they tried to apply the techniques they learned to reduce symptoms or things the therapists had said or done to real situations in their everyday lives. In addition, they underscored the well-known importance of having agreed goals. However, the patients in the present study also reported that the very act of receiving therapy was something they had to learn. They did not always know what the therapists expected from them or why there might be periods of silence. Improvement and satisfaction with the therapy seemed closely related to whether such frustrations were addressed and resolved. Thus, the obstacles in therapy in the present study were also related to the situation or context in which the relationship between the patient and the therapist evolved. It seems important, then, to consider that what facilitates improvement in therapy may sometime relate more to the situation than to relational factors. The patients’ view of themselves, their therapists and the situation of being in therapy should all be considered in the therapy process.

Generally, as argued by Timulak and Keogh [54], what is helpful for one patient may be unhelpful for another, and the same helpful factors may be helpful in contrary ways for different patients. Each patient will have his or her own context—culturally, personally or valuably—according to which the patient will understand his or her life and therapy. Thus, when making efforts to improve psychotherapy it seems crucial to develop patient feedback systems that are sensitive to individual factors and preferences, such as those described in the present study. This is true even if these preferences differ from what qualitative and quantitative evidence has shown to be helpful and important in psychotherapy to this point.

### 4.4. Limitations and Strengths

A hermeneutic approach is not suitable for developing generable, nomothetic knowledge that corresponds to contexts other than the one in which it originated. Its strength lies in its ideographical perspective, focusing meaningful and coherent descriptions of events that, in principle, cannot occur again. Hence, our results do not apply directly to other individual patients, and are not statistical generalizable. Rather, they express what some patients with depression, and potentially other similarly diagnosed patients, might benefit from in therapy. The present study did not address the complex theoretical question of how, or from where, experiences originate and come into being. We assume that patients would likely have nuanced or highlighted other aspects of their therapy experiences if they were to share these experiences with other people in other contexts and circumstances. This must be considered when applying the results of the present study to other patients in other clinical settings with other therapeutic interventions or psychiatric disorders. Hence, our results may fail if used to facilitate the therapy of other patients if they are not situated specifically for those patients’ circumstances and ways of interpreting their individual situations. Reporting only the helpful aspects of PDT and leaving out unhelpful experiences might give a biased impression of the “superiority” of PDT. This is unintended. Hypothetically, the patients we studied might have experienced other therapy modes as equally helpful. Additionally, our results do not provide any information about the patients’ degree of improvement or satisfaction with the therapy. Although the present study focused on intra-therapeutic factors, we recognize that improvement is multifactorial and includes extra therapeutic factors, such as support from family and friends or getting a job [55,56]. A strength of the study is the amount and space given to the patients utterances described in an empirical and patient near tone. This was done without viewing their experiences through specialized theoretical lenses or concepts. As such, we believe this study provides insights into how patients themselves might feel they improve from depression.

## 5. Conclusions

The present study explored ten adult patients’ experiences of improvement in time-limited PDT. The patients experienced improvements by opening up, taking care of themselves and demonstrating agency. They experienced the therapists as helpful through the therapists’ questions and the pressure that followed and when they gave the patients support or advice for dealing with everyday life. The patients improved as they gained new perspectives and understandings of themselves, increased their self-awareness and changed their ways of thinking and feeling. They achieved this through agreed goals and learnings from therapy, after gradually learning how to receive therapy. The patients seemed to experience the helpful therapy as a dynamic whole, in which a focus on symptoms and intrapsychic functioning was combined with a focus on the challenges they faced in life outside of therapy.

## Figures and Tables

**Table 1:** Dimensions and themes promoting improvement in time limited psychodynamic psychotherapy (PDT) for depression.

Dimensions	Themes
Therapist activities	Supporting and acknowledging
	Offering advice and tips for everyday life
	Questioning and pressuring
Patient activities	Opening up
	Caring for oneself
	Showing agency
Facilitators	Learnings from therapy
	Learning to receive therapy
	Agreed goals
Achievements	New perspectives and understanding
	Increased self-awareness and mastery
	Changed thinking and feeling
